# Postural adjustments impairments in elderly people with chronic low back pain

**DOI:** 10.1038/s41598-021-83837-2

**Published:** 2021-02-26

**Authors:** Daniela Rosa Garcez, Gizele Cristina da Silva Almeida, Carlos Felipe Oliveira Silva, Tainá de Souza Nascimento, Anselmo de Athayde Costa e Silva, Ana Francisca Rozin Kleiner, Givago da Silva Souza, Elizabeth Sumi Yamada, Bianca Callegari

**Affiliations:** 1 University Hospital Bettina Ferro de Souza, Federal University of Pará, R. Augusto Corrêa, n1, Belém, Pará 66075-110 Brazil; 2 Neuroscience and Cell Biology Graduate Program, Federal University of Pará, R. Augusto Corrêa, n 1, Belém, Pará 66075-110 Brazil; 3 Laboratory of Human Motricity Sciences, Federal University of Pará, Av. Generalíssimo Deodoro 01, Belém, Pará 66050-160 Brazil; 4 Tropical Medicine Center, Federal University of Pará, Av. Generalíssimo Deodoro 92, Belém, Pará 66050-240 Brazil; 5 Master’s Program in Human Movement Sciences, Federal University of Pará, Av. Generalíssimo Deodoro 01, Belém, Pará 66050-160 Brazil; 6 Department of Physiotherapy, Federal University of São Carlos, Rodovia Washington Luiz km235, caixa postal 676, São Carlos, São Paulo 13565-905 Brazil; 7 Graduate Program in Medical Sciences and Oncology, Federal University of Pará, Rua dos Mundurucus 4487, Belém, Pará 66073-005 Brazil; 8 Instituto de Ciências da Saúde, Avenida Generalíssimo Deodoro, nº1, Belém, Pará 66055-240 Brazil

**Keywords:** Geriatrics, Motility, Muscle contraction

## Abstract

Chronic low back pain (CLBP) is associated with postural control impairments and is highly prevalent in elderly people. The objective of this study is to verify whether anticipatory postural adjustments (APAs) and compensatory postural adjustments (CPAs) are affected by CLBP in elderly people by assessing their postural control during a self-initiated perturbation paradigm induced by rapid upper arm movement when pointing to a target. The participants’ lower limb muscle onset and center of pressure (COP) displacements were assessed prior to perturbation and throughout the entire movement. T_0_ moment (i.e., the beginning of the movement) was defined as the anterior deltoid (DEL) onset, and all parameters were calculated with respect to it. The rectus femoris (RT), semitendinosus (ST), and soleous (SOL) showed delayed onset in the CLBP group compared with the control group: RF (control: − 0.094 ± 0.017 s; CLBP: − 0.026 ± 0.012 s, t = 12, p < 0.0001); ST (control: − 0.093 ± 0.013 s; CLBP: − 0.018 ± 0.019 s, t = 12, p < 0.0001); and SOL (control: − 0.086 ± 0.018 s; CLBP: − 0.029 ± 0.015 s, t = 8.98, p < 0.0001). In addition, COP displacement was delayed in the CLBP group (control: − 0.035 ± 0.021 s; CLBP: − 0.015 ± 0.009 s, t = 3; p = 0.003) and presented a smaller amplitude during APA COP_APA_ [control: 0.444 cm (0.187; 0.648); CLBP: 0.228 cm (0.096; 0.310), U = 53, p = 0.012]. The CLBP group required a longer time to reach the maximum displacement after the perturbation (control: 0.211 ± 0.047 s; CLBP 0.296 ± 0.078 s, t = 3.582, p = 0.0013). This indicates that CLBP elderly patients have impairments to recover their postural control and less efficient anticipatory adjustments. Our results suggest that people with CLBP have altered feedforward hip and ankle muscle control, as shown from the SOL, ST, and RT muscle onset. This study is the first study in the field of aging that investigates the postural adjustments of an elderly population with CLBP. Clinical assessment of this population should consider postural stability as part of a rehabilitation program.

## Introduction

Low back pain (LBP) is one of the most frequent symptoms reported by older people. It is defined as any pain or discomfort between one’s last ribs and the lower gluteal line, with or without irradiation symptoms to the lower limbs^1^. Pain duration is one of the criteria for classifying LBP types. Long-duration pain is defined as chronic low back pain (CLBP)^2^. The most typical classification of CLBP is nonspecific CLBP, which refers to cases in which the etiology of a patient’s pain is unidentifiable^3^. In addition, CLBP is a risk factor for incapacity and invalidity^3,4^; it is the leading cause of functional limitations associated with the performance of daily living activities^3,4^, with a higher prevalence in women aged 60–69 years^5^. In fact, aging and CLBP are important factors that affect an individual’s postural control^6–8^.

Postural control is necessary to maintain one’s center of mass within the basis of support, preventing falls. When perceiving an upcoming perturbation that may result in center of mass (COM) displacement, our central nervous system uses anticipatory postural adjustments (APAs) and compensatory postural adjustments (CPAs)^9,10^. Hence, when individuals execute voluntary movements that generate self-initiated perturbations, APAs are triggered before their movements begin to prevent or minimize any effects from such perturbation^10,11^. This is observed during rapid arm movement, such as in a pointing task at maximum velocity. APAs are represented by postural muscle activations beginning from − 150 to − 100 ms prior to the focal movement, in a feedforward centrally programmed mechanism^12^. CPAs are a feedback-based control mechanism that restore balance through muscle activation following perturbation^7,10^. Center of pressure (COP) displacements are classically described to access APAs (measured as the onset of COP displacement and its amplitude prior to perturbation) and CPAs (described as the recovery time necessary to set back to the initial position, and the COP amplitude after perturbation)^7,8,13,14^.

Studies have reported an important relation between APAs and CPAs, indicating that the greater the anticipatory adjustments, the less necessary are the compensatory adjustments to maintain one’s stability^7,10^.

Aging affects postural control, increasing both the risk of falls and the fear of falling^15^. Previous studies have reported that the elderly population presented the delayed onset of postural muscles during APAs^7,14,16^, decreased APAs and/or increased CPAs postural muscle activation^7^, different muscle patterns or strategies to maintain posture^8,14,17^, and delayed COP onset during APAs, when compared with young people^8,14^.

Postural control in people with CLBP has been described in the literature, albeit with inconclusive evidence. Most studies are primarily focused on the trunk musculature and young population, using self-initiated perturbation in the upright posture^6,18^. Differences in spinal erector muscle were investigated between healthy and CLBP adults. Some studies indicated a delay in the onset of this muscle^18,19^ in CLBP individuals, whereas others showed no differences between groups^20^. A recent systematic review reported that the onset of abdominal muscles during APAs was delayed in the CLBP population when compared with healthy controls^6^. However, the authors stated that evidence other than muscle latency, such as those based on measures from force platforms or kinematics, are not available in the literature. Once changes caused by CLBP in APAs onsets affect the movements and forces to which one’s trunk is exposed during an activity, its functional consequences remain unclear^21^. In addition, only a few APA studies in individuals with CLBP included investigations pertaining to lower limb muscles^22,23^.

Hence, the literature provides limited evidence regarding changes in postural control in CLBP patients. Moreover, such studies excluded the elderly population and, therefore, do not provide information regarding postural control in older people with LBP. Evaluating postural strategies and balance is paramount when assessing and rehabilitating elderly patients, since they pose a high risk of falling, particularly when performing daily life activities.

This study aims to investigate the differences in APAs and CPAs in elderly people with and without CLBP by assessing their postural control after a self-initiated perturbation paradigm. We hypothesize that elderly people with CLBP will present delayed and increased COP displacements during the APA phase owing to a higher COP displacement during the CPA period. This implies that they will require more time to restore postural control. Additionally, we hypothesize that muscle onset during APAs will be delayed in elderly people with CLBP.

## Materials and methods

The cross-sectional observational study performed in this investigation was approved by the Ethics Committee of the Federal University of Pará (protocol #25317119.4.0000.0018) as well as the Observational Studies in Epidemiology (STROBE) Statement. Written informed consent was obtained from all participants before the study was started. The study was performed from March 2019 to March 2020.

### Participants

Thirty elderly participants participated in the present study. They were segregated into two groups, matched by age, height, and weight: CLBP (n = 15; 4 males; 11 females) and control (n = 15; 3 males; 12 females). The elderly with CLBP were submitted to a geriatrician-confirmed diagnosis of nonspecific CLBP, of which the inclusion criteria were as follows: (1) history of chronic unilateral or bilateral CLBP (≥ 3 months) without pain referral to their lower limbs; (2) the ability to stand and walk independently; (3) having a score ≤ 2 on the numeric pain rating scale (NRS)^13,24,25^; and (4) the ability to understand verbal commands for executing the required tasks^26,27^. The exclusion criteria were nonmechanical CLBP (e.g., fracture, malignancy, and infection); radicular signs; history of back surgery; diagnosis of inflammatory joint disease; severe osteoporosis; metabolic or neuromuscular diseases; other chronic pain pathologies; any major circulatory, respiratory, neurological, or cardiac diseases; or cognitive deficit.

For the control group, the inclusion criteria were not having CLBP throughout the previous year or back pain lasting longer than one week in the previous 3 years. The exclusion criteria were previous histories of neurological or musculoskeletal disorders that induced visible gait abnormalities. To better characterize the sample, both groups completed the Oswestry disability index (ODI)^28^ for assessing function disability. Table 1 summarizes the main characteristics of the participants from both groups.

**Table 1:** Sample characteristics.

	CTL group	CLBP group	p-value
Age (years)	70.2 ± 4.6	70.13 ± 6.5	0.9745
Height (m)	1.56 ± 0.04	1.55 ± 0.03	0.7935
Weight (kg)	54.07 ± 2.21	54.53 ± 2.19	0.5676
NRS	–	1.53 ± 0.63	–
ODI (%) #	0 (0; 4)	12.5 (10; 16)	< 0.0001

Data expressed as mean ± SD if presented with a normal distribution, or median and percentiles if presented with nonparametric distribution.

The level of significance was 0.05.

*NRS* numeric pain rating scale, *ODI* Oswestry disability index.

### Experimental setup for APA assessment

**Figure 1:**
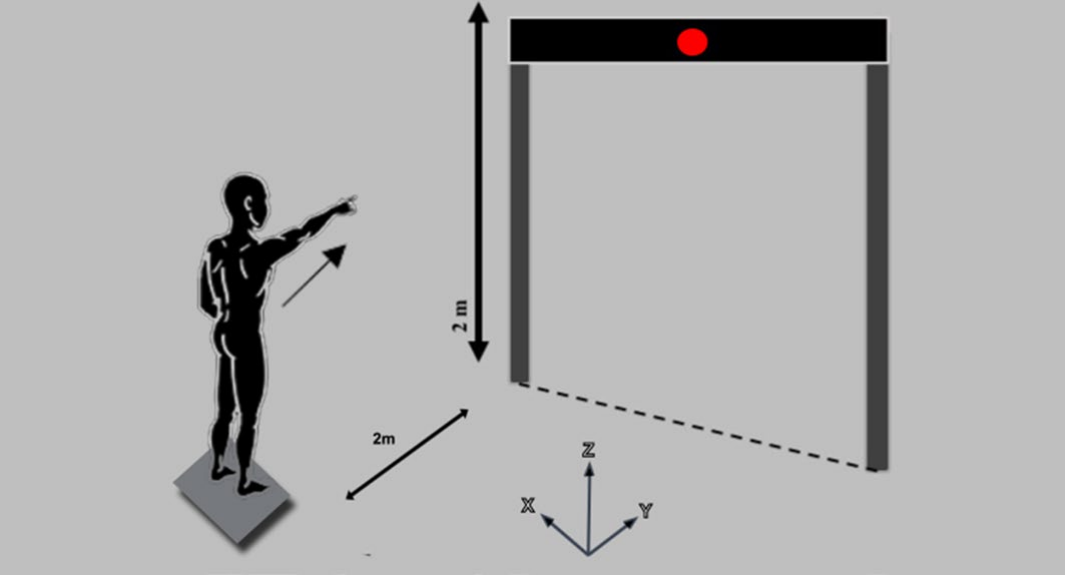
Experimental setup for task showing a participant in final posture. Central diode of the bar was placed exactly in front of participants’ right shoulder. Participants were asked to point their index finger, with extended elbow, at the central diode whenever it was turned on.

The participants stood with bare feet on a force platform and were instructed to use a self-selected pleasant position such that the mid-point of their heels were separated by a distance equal to the width of their shoulder, with feet externally rotated up to 15°, which is considered a natural comfortable position. They were instructed to observe a horizontal bar that was placed in front of them, 2 m above the floor, and 2 m away from the participants’ feet, with a light-emitting diode (LED) aligned to their right shoulder. The participants stood with their arms relaxed down along their bodies, and their right index finger pointing to the ground. Subsequently, they were asked to move the arm, using their right index fingers to point the LED every time it was turned on in a ten-trial round. Electrical activity in the anterior deltoid (DEL) was visually verified prior to switching on the LED to ensure that the deltoid muscle was relaxed. For every trial, the participants were instructed to extend their elbows and raise their arms as fast as possible after perceiving the visual stimuli, maintaining their arm in the air for a few seconds and then moving it back to the initial position (Fig. 1).

### Kinetic, kinematic, and electromyographic recording

In this study, a force platform (Biomec 400-041, EMG System) sampled at 100 Hz was used to record three-dimensional ground reaction forces. Using the obtained data, we computed the coordinates of the participants’ COP in the anteroposterior direction. A three-dimensional motion analysis system (Simi Motion, Simi, Germany) with three cameras at a sampling frequency of 120 Hz was used to record the participants’ movements. Each of them comprised four infrared reflective markers placed at the main joints of their right upper limb (i.e., index, wrist, elbow, and shoulder). Surface electromyographic (EMG) data were recorded from the participants’ dominant-side leg muscles: tibialis anterior (TA), soleus (SOL), rectus femoris (RF), semitendinosus (ST), and anterior deltoid (DEL), using an EMG device (Emgsys 30,306, EMG System do Brasil, Brazil), with a sampling rate of 2 kHz and a frequency spectrum of 20–500 Hz. The EMG signals were amplified (4000) and digitized with a 16-bit resolution. The participants’ skin was prepared for the placement of Ag/AgCl electrodes (Medtrace 200-Kendall, Canada) using Nuprep (Weaver and Company, Aurora, United States) and an alcohol-based sanitizer. By following the recommendations of the Surface Electromyography for Non-Invasive Assessment of Muscles guidelines^29^, we placed active electrodes on their muscles at 20 mm intervals and the reference electrode on their right fibular malleolus.

### Data analysis

Raw EMG signals were bandpass filtered between 20 and 400 Hz, full-wave rectified, and bidirectionally filtered using a 6 Hz low-pass, second-order, zero-lag Butterworth filter. Muscle onset (concerning both the activation/inhibition of a muscle) was detected in relation to T_0_ via visual inspection performed by two blinded examiners. The low-pass filtering generated a smooth envelope, which, in combination with the raw signal, was used for the visual identification of the muscle onsets. T_0_ moment (i.e., the beginning of the movement) was defined as the onset of DEL. After the onset of each trial, we calculated the timing of each muscle activation with reference to the DEL onset^16,18^.

Triggers of kinematics and force platform data were provided to two channels of the EMG to permit data synchronization and offline analysis using MatLab programs (MathWorks, Natick, MA, USA). Ten trials were performed for the calculations. The kinematic parameters of the participants’ arm movements extracted by the index finger (trajectory and tangential velocity profile) were analyzed. The raw coordinate data on the x-, y-, and z-axes were generated from the video analysis and then filtered using a bidirectional 10-Hz low-pass, second-order Butterworth filter. The kinematic variables were (1) the reaction time (RT), measured as the time interval from the LED stimuli to T_0_ moment; (2) the peak velocity (PV): the maximum velocity reached by a participant’s arm during the pointing task; (3) the time to peak velocity (TPV), measured as the time from the T_0_ moment to the maximum peak velocity moment; (4) the total movement duration (MD): the time interval between the T_0_ moment and the end of the trial when a participant’s index finger stops pointing to the diode (velocity returns to zero); and (5) the index tangential velocity profile, which was calculated by the ratio of acceleration time (i.e., the fraction of movement time required to reach peak velocity) to the total movement duration (ACC/MD). This velocity profile is classically described as reflecting the content of motor planning^30^. More specifically, asymmetries in the relative acceleration duration (i.e., ACC/MD) demonstrate different motor plans for the execution and control of arm movements.

**Figure 2:**
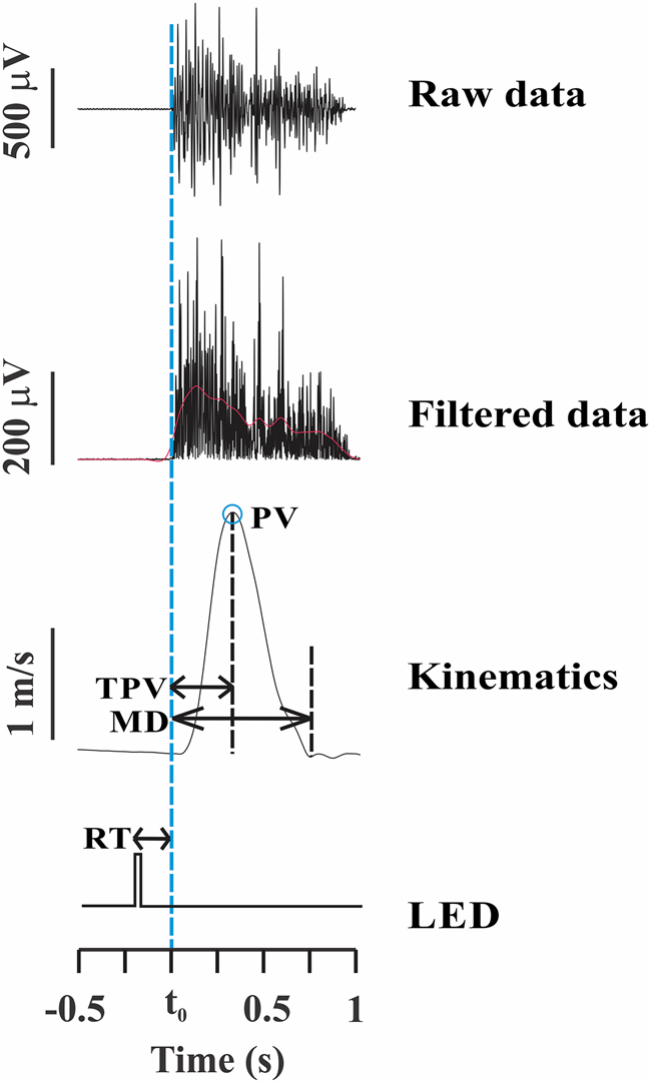
DEL onset detection after LED stimuli and using kinematic parameters. Dashed blue line represents T_0_ moment of the task.

Figure 2 presents the DEL onset detection after the LED stimuli and the kinematic parameters.

**Figure 3:**
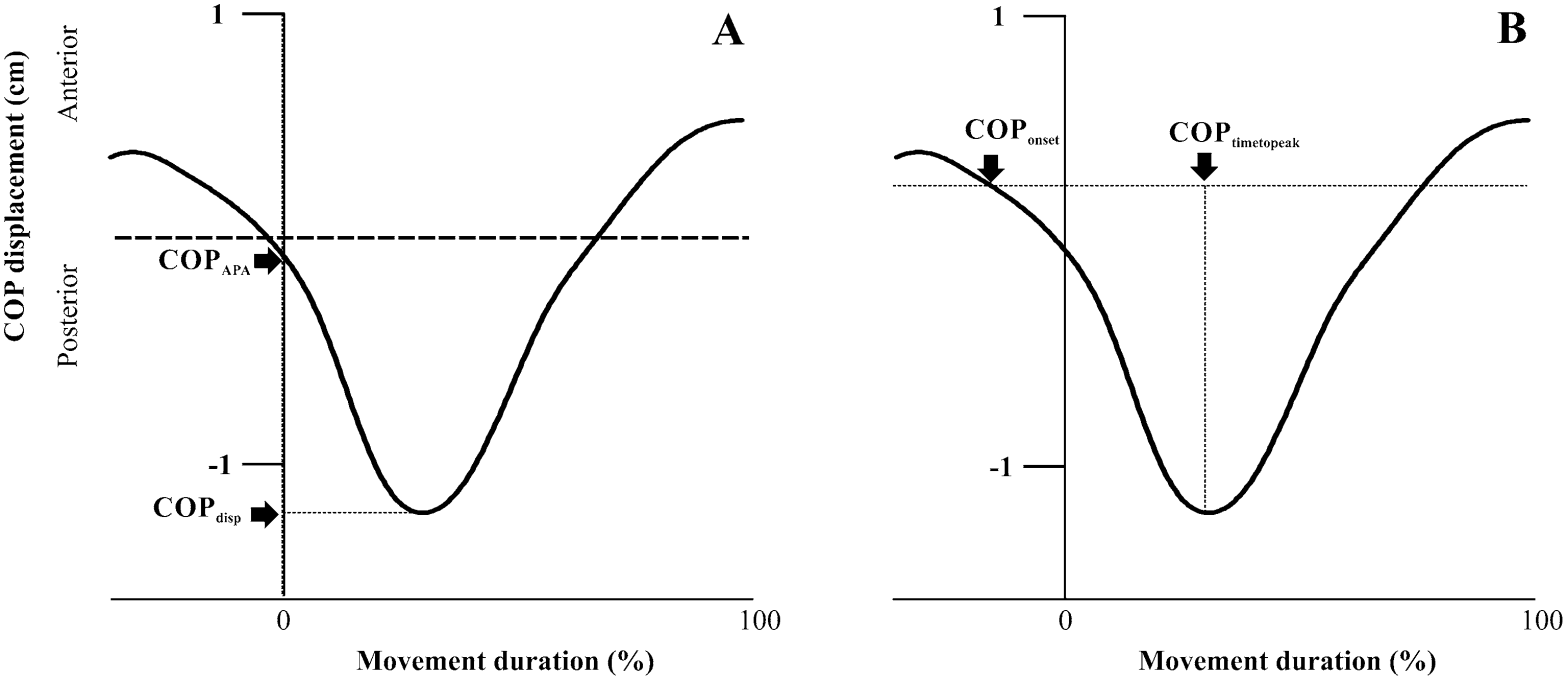
(**A**, **B**) Anteroposterior COP displacement (y-axis) when a participant moves his/her arm. Dashed line represents movement onset. Four variables: (1) COP_APA_, amplitude of backward COP displacement at T_0_; (2) COPdisp, maximum backward displacement after T_0;_ (**A**); (3) COPonset, time of initial backward displacement before T_0_; and (4) COPtimetopeak, time to reach maximum displacement (**B**).

COP displacements in the anteroposterior direction were calculated based on previous studies^31^. The baseline activity used for the calculation was from − 500 to − 400 ms in relation to the T_0_ moment. The displacement in the anteroposterior COP dimension was analyzed and four variables were derived from it. The first two variables were anticipatory in nature, whereas the other two variables were compensatory in nature: (1) the beginning of the COP displacement before T_0_ moment, measured as the time when the COP displacement was smaller than the mean of its baseline value plus 2 standard deviation (SD) (COP_onset_) (Fig. 3B)^13^; (2) the anteroposterior COP displacement at T_0_ moment (measured from the baseline amplitude), which is anticipatory in nature, known as the amplitude of the COP displacement at T_0_ (COP_APA_) (Fig. 3A); (3) the peak displacement measured, which is compensatory in nature (COP_disp_), as the maximum displacement after T_0_ moment (Fig. 3A); and (4) the time to reach this peak maximum displacement (COP_timetopeak_) (Fig. 3B)^7,8,14^.

### Statistical analysis

GraphPad Prism 6 (San Diego, California, USA) and BioEstat 5.3 (Belém, Pará, Brazil) for Windows was used to perform the statistical procedures of this study. The data distribution was tested via the Shapiro–Wilk test. Medians with first and third quartiles were reported for nonparametric outcomes, whereas the mean and SD for parametric outcomes. Data were compared between groups. Unpaired t-test was performed to compare the parametric outcomes (age, height, weight, muscles onset during APAs, COP_onset_, and COP_timetopeak_; and the kinematic parameters PV, MD, RT, and ACC/MD), whereas the Mann–Whitney test was performed to compare nonparametric outcomes (ODI score, time to peak velocity, CO_APA_, and COP_disp)_. For all these statistical treatments, the significance level was p ≤ 0.05.

## Results

### Kinematic characteristics

Table 2 summarizes the pointing task kinematic parameters. In this study, no statistical differences were observed between groups in terms of kinematic features.

**Table 2:** Comparison between kinematic parameters.

	Control group	CLBP group	p-value
Reaction time (s)	0.160 ± 0.031	0.177 ± 0.068	0.407
Peak velocity (m/s)	5.375 ± 1.44	4.709 ± 1.062	0.161
Acceleration time/movement duration	0.462 ± 0.095	0.460 ± 0.060	0.959
Time to peak velocity (s)	0.348 (0.295; 0.377)	0.315 (0.290; 0.353)	0.290
Movement duration (s)	0.669 ± 0.080	0.676 ± 0.092	0.815

Data expressed as mean ± SD if presented with a normal distribution, or median and percentiles if presented with nonparametric distribution.

### Muscle latency

**Figure 4:**
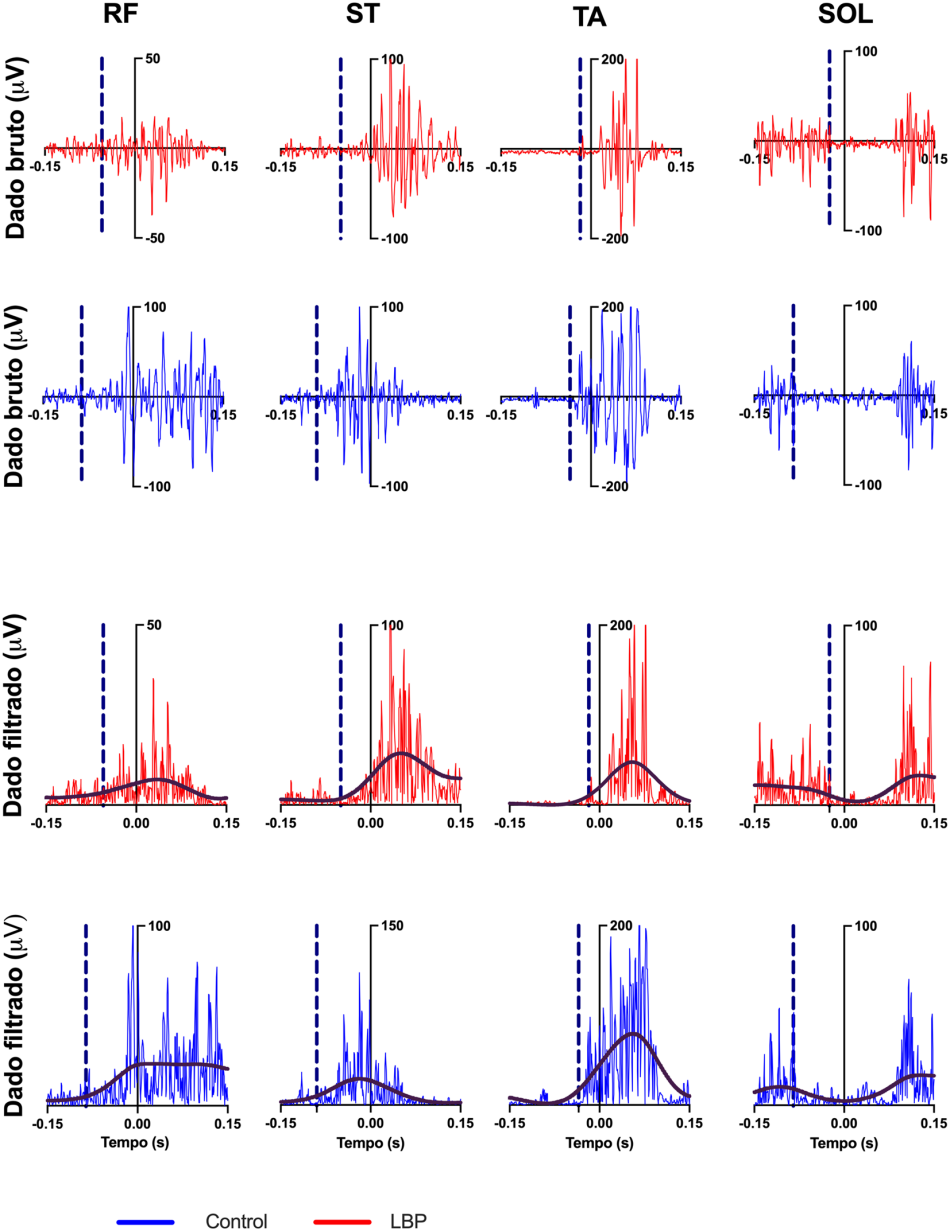
Raw and rectified 6 Hz low-pass filtered muscles activity of a typical participant of each group, recorded during one single trial. Vertical blue dashed line indicates muscles onset (t_0_). Muscle abbreviations: *ST* semitendinosus, *RF* rectus femoris, *SOL* soleous, *TA* tibialis anterior. Control participants’ anticipation compared with CLBP results.

Figure 4 presents each muscle onset of a single trial of one control elderly participant and one CLBP elderly participant.

**Figure 5:**
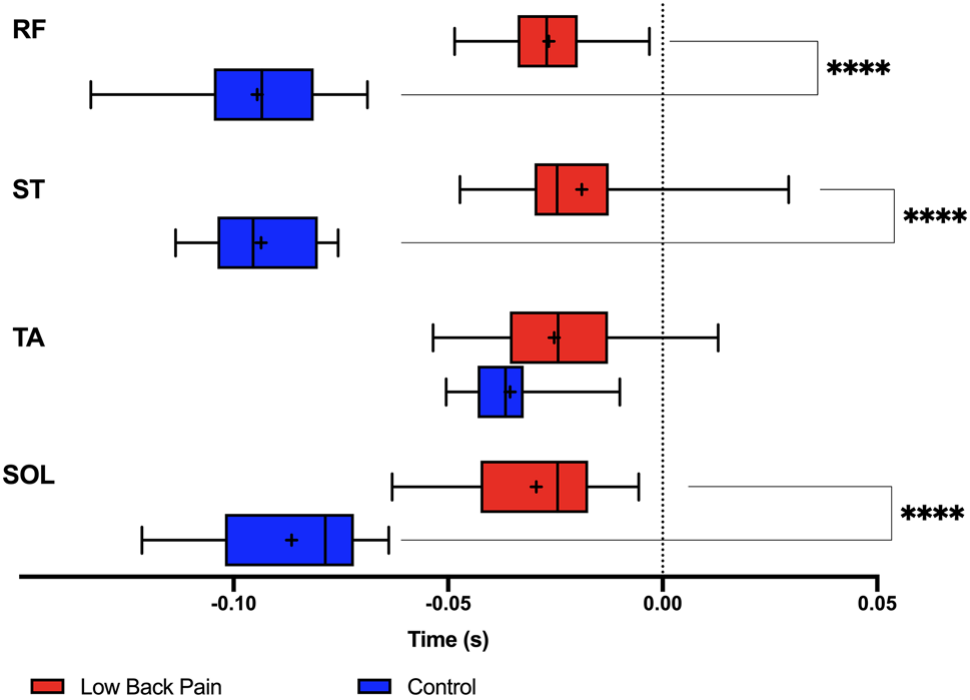
Muscle activity onsets for both control elderly and CLBP elderly participants. Muscle abbreviations: *RF* rectus femoris, *ST* semitendinosus, *SOL* soleus, *TA* tibialis anterior. Differences in latencies were significant when p < 0.05 (*). Data expressed by central line = median, box = 25 and 75 percentiles, and whiskers = min and max values (mean values inside box marked as X).

Proximal muscles (RF and ST) and the distal muscle (SOL) showed delayed onset in the CLBP group when compared with the control group (Fig. 5). The onset of APA activity was as follows: RF (control: − 0.094 ± 0.017 s; CLBP: − 0.026 ± 0.012 s, t = 12, p < 0.0001); ST (control: − 0.093 ± 0.013 s; CLBP: − 0.018 ± 0.019 s, t = 12, p < 0.0001); TA (control: − 0.035 ± 0.009 s; CLBP: − 0.025 ± 0.017 s, t = 1.96, p = 0.059); and SOL (control: − 0.086 ± 0.018 s; CLBP: − 0.029 ± 0.015 s, t = 8.98, p < 0.0001).

### Displacements of COP

**Figure 6:**
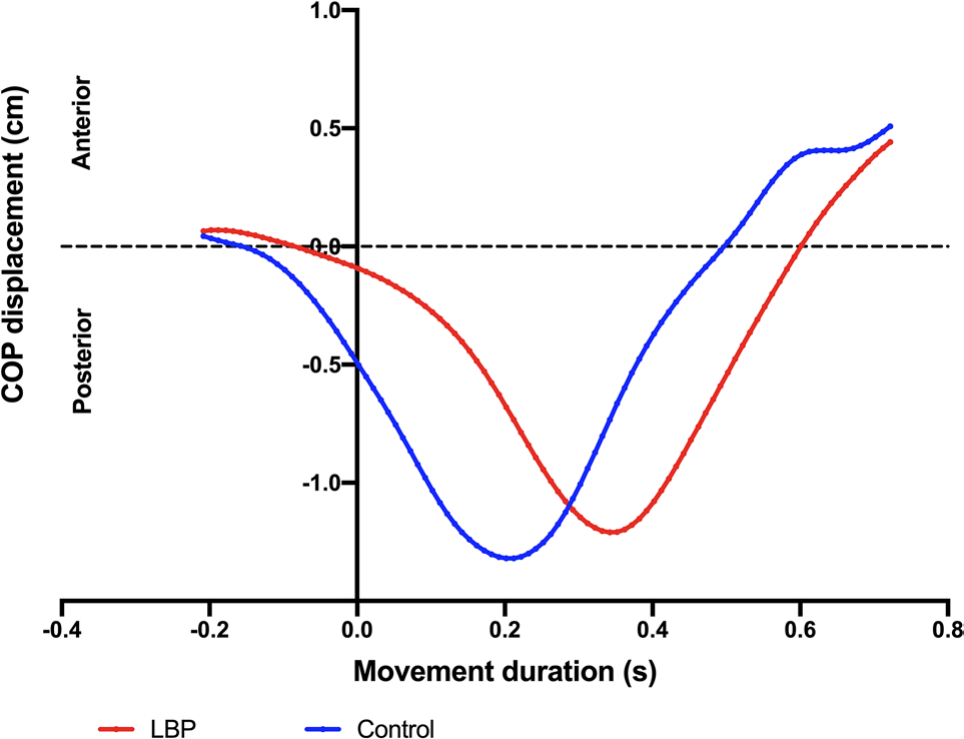
Anteroposterior COP displacement during arm movement of a typical participant recorded during one single trial. CLBP participants presented an earlier COP_onset_, a higher amplitude in COP_APA_, and less time to reach the maximum displacement after T_0_ (lower COP_timetopeak_). No differences was observed between groups in COP_disp_.

Figure 6 presents the anteroposterior COP displacement of a single trial of one control elderly participant and one CLBP elderly participant.

**Figure 7:**
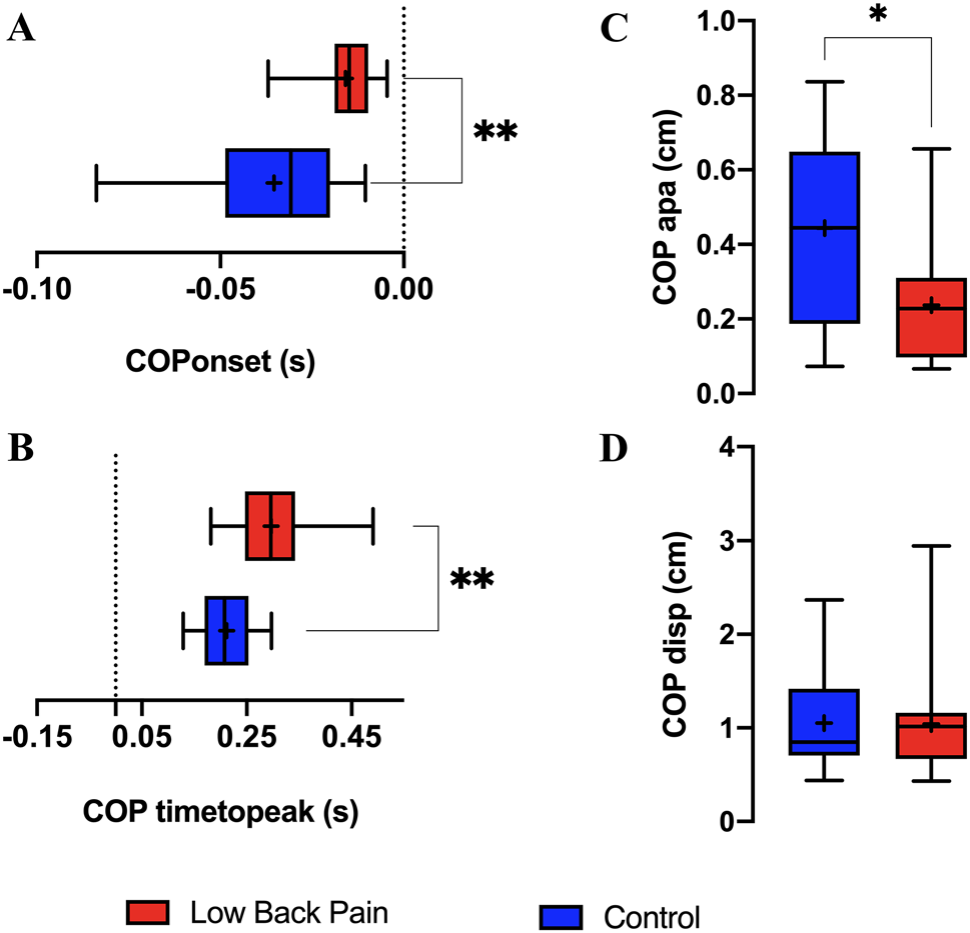
(**A**) COP_onset;_ (**B**) COP_timetopeak;_ (**C**) COP_APA_, and (**D**) COP_disp_. Differences were significant when *p* < 0.05 (*). Data expressed by central line = median, box = 25 and 75 percentiles, and whiskers = min and max values (mean values inside box marked as X).

Figure 7 depicts the COP_onset_ (A)_,_ COP_timetopeak_ (B), COP_APA_ (C), and COP_disp_ (D). The CLBP group showed delayed COP_onset_ (control: − 0.035 ± 0.021 s; CLBP: − 0.015 ± 0.009, t = 3; p = 0.003) and a smaller COP_APA_ (control: 0.444 cm (0.187; 0.648); CLBP: 0.228 cm (0.096; 0.310, U = 53, p = 0.012) compared with the control group (Fig. 7A,C). Although both groups reached a similar COP_disp_ after perturbation [control: 0.849 cm (0.703; 1.418); CLBP: 1.013 cm (0.666; 1.162), U = 105, *p* = 0.766)], the CLBP group required more time to reach it, presenting a higher COP_timetopeak_ compared with the control group (control: 0.211 ± 0.047, CLBP 0.296 ± 0.078 s, t = 3.582, p = 0.0013) (Fig. 7B,D).

## Discussion

The study was conducted to investigate the effects of CLBP on APAs and CPAs in elderly people during a self-initiated perturbation paradigm. In general, our results demonstrated a delayed activation of the lower limb proximal muscles (RF and ST) and the distal muscle (SOL) in elderly patients with CLBP compared with the match-aged control elderly. During the APA phase, the COP displacement delayed and presented a smaller amplitude in the CLBP elderly. No differences was observed in the peak of COP displacement during the CPA period. However, the CLBP elderly required more time to reach it. Since a longer time for stabilizing posture is associated with poor postural control^32^ and impaired ability to recover postural stability throughout the movement (from the beginning through the end of one’s arm movement)^13^, the CLBP elderly patients have exhibited less efficient anticipatory adjustments and greater difficulty in recovering postural control during the compensatory phase.

### Kinematic characteristics

Both groups presented similar kinematic features, demonstrating that they fully performed the task similarly, i.e., with the same perturbation magnitude. Studies regarding the pointing task paradigm demonstrated that a person’s postural adjustments depended on velocity: the higher the speed, the worse was the postural adjustments^8,17^. The glenohumeral joint was subjected to opposing forces when the direction of shoulder motion was changed; acceleration and deceleration were involved when performing movement tasks. Accelerations caused by rotation forces in this joint resulted in multipoint reaction forces that may perturb other parts of an individual’s body^33^. Hence, kinematic characteristics, such as limb acceleration peak^13^, mean speed^17^, or velocity peak^8^ of one’s upper limb are typically used to guarantee similar perturbation magnitudes. Furthermore, our results indicate that differences in postural adjustments between groups may have CLBP as a determining factor, rather than differences in the movement performed.

### Altered muscular patterns in CLBP elderly

The results of the ST, RF, and SOL muscle onsets were consistent with our hypothesis, since the CLBP elderly patients presented delayed activation when compared with the control elderly participants. Some studies have previously reported delayed muscle activation related to CLBP during self-initiated perturbation in the upright posture; however, most of them primarily focused on young participants’ trunk muscles^6,18,19^. A recent meta-analysis revealed a significant and substantial heterogeneity in muscle onsets^6^, stating that the onset of anterior trunk muscles (i.e., transverse abdominis, internal oblique, external oblique, and rectus abdominis) was delayed in CLBP participants when compared with healthy controls, with an acceptable amount of heterogeneity. Meanwhile, studies concerning posterior trunk muscles (i.e., spinal erectors) were controversial. Some outcomes indicated delayed muscle onset^18^ in CLBP individuals, whereas others showed no difference between groups^18,20,24,34^. Studies regarding lower limb muscles are scarce, thereby limiting the comparison of their results with ours. To our knowledge, only three related studies have been performed, among which one included the TA, RF, and GAS^35^, whereas the others included the ST^23^ and no GAS^25^. Sadeghi et al.^25^ discovered delayed activation of the trunk muscles in patients with CLBP; earlier activation of GAS was present in this group, and the authors speculated that CLBP patients adopted the ankle strategy^22^ more frequently toward postural disturbance than healthy participants. Because that study included no other lower limb muscles, it was difficult to extend its results to ours.

Delayed muscle onset is also described in the literature as a dysfunctional joint strategy^35^: delayed TA muscle activation in patients with nonspecific CLBP may occur when they perform challenging tasks, suggesting a dysfunctional ankle strategy in this population. Our results support this premise, since, in this study, the CLBP participants presented delayed SOL activation compared with the control participants.

Only one study^22^ investigated the differences between people with CLBP and healthy individuals with ST. Even though the results showed that the ST was delayed in CLBP participants, we cannot compare them with our ST findings because the authors did not investigate postural perturbation (i.e., the participants were performing a hip extension in prone).

In summary, our findings suggest that CLBP participants have altered feedforward motor control in the hip and ankle muscles, as demonstrated by delayed onsets of the ST, RF, and SOL. These findings are consistent with previously described strategies for young people with CLBP, such as decreased hip strategy^36^, increased trunk co-contraction activity^37^, and altered activation time of axial postural muscles^6,18,24,34^.

### Impaired COP control in CLBP elderly

Our COP results partially confirmed our hypothesis, as we observed delayed COP_onset_ and reduced COP_APA_ in CLBP elderly patients when compared with the control group. By analyzing the participants before performing the arm-moving task, we observed a backward displacement of COP in both groups. Rapid bilateral or unilateral upper limb flexion generated self-perturbation, in which a forward center of mass (COM) displacement resulted in a backward displacement of the COP^38^. This occurred prior to perturbation and continued throughout the compensatory phase until postural control was recovered, as indicated in our results^8,39^.

Previous studies used COP_onset_ and COP_APA_ to assess the quality of APAs; it was reported that the smaller the onset and amplitude of the COP, the less efficient was the preparation for the expected perturbation^7,8^. Therefore, as hypothesized, the control group delivered more efficient APAs when compared with the CLBP elderly patients.

Contrary to the expected results, no differences was observed between groups in terms of the COP_disp_ after disturbance. However, the time required to reach it was higher in the CLBP elderly participants. The maximum displacement of COP after perturbation and the time to reach this peak guided the interpretations of compensatory adjustments after postural disturbance^7,8,13,40^. In general, it has been reported that smaller COP excursions after perturbation characterize better postural recovery^7^. We anticipated this result from the control group instead of the CLPB group; however, the phenomenon did not occur. Some authors have demonstrated a similar or even lower maximum COP displacement in adults with CLBP when compared with healthy participants^13,40,41^. According to them, in contrast to healthy individuals, CLBP participants may avoid activating muscles that are required to create specific body movements and forces. In fact, it indicates a constraint during the recovery period after voluntary arm movement to prevent falling. This results in a smaller COP excursion, decreasing the driving force to return to equilibrium^42^. This may explain why differences were not observed between both groups. Meanwhile, the time to reach the peak of COP displacement was significantly shorter in the control elderly group, indicating that the healthy elderly participants were able to recover their balance faster than those with CLPB. This outcome is consistent with findings previously reported in adults with and without CLBP^13,40^, as it is well known that increased time for postural stabilization is associated with poor postural control^13,32,40^. Therefore, our COP results demonstrate a reduction in the quality of postural recovery in elderly individuals with CLBP.

Some theoretical models support our findings related to muscles and COP. First, patients with CLBP have an altered proprioception in their lumbar-pelvic region, which resulted in difficulties in the calculations of the initial or final positions of body segments or in reproducing a previously set position^43^. Owing to the lack of feedback from their lumbar spine (i.e., the spine position was uncertain), CLBP patients presented ineffective control of their COM position to use the hip strategy in postural control (i.e., when lumbo–pelvic movement was involved). The hip strategy is complex and requires the interpretation of angle changes at the hip and spine to calculate the COM position^44^. This may explain why the CLBP elderly individuals performed worse when using the hip strategy^36^.

Altered postural muscle control was described in CLPB participants as a mechanism to minimize trunk motion and maintain protective stiffness to avoid pain or successive injuries^43,45,46^. Moreover, the fear of falling, typically present in elderly people, may alter muscle control in a protective manner^32^. An altered timing of muscle activation and increased co-contraction in the axial muscles during body perturbation has been reported in individuals with CLBP^25,34,37,45^. This increases spinal stiffness and reduces spinal movement^47–49^.

Finally, when performing rapid voluntary arm movements, the CLBP elderly participants presented impaired APAs when compared with the control group. Delayed lower limb proximal and SOL muscles and COP onset highlighted this condition. In addition, their inability to achieve timely postural recovery (i.e., higher COP_timetopeak_) reflected delayed postural control during the compensatory phase. Hence, elderly individuals with CLBP might encounter a higher risk of falling under situations that require rapid recovery. Clinical assessment for this population should account for postural stability during rehabilitation programs.

## Supplementary information

Supplementary Legend.

Supplementary Information.

## Data Availability

Data available as “Supplementary files”.
