# Author Correction: Postural adjustments impairments in elderly people with chronic low back pain

**DOI:** 10.1038/s41598-021-96105-0

**Published:** 2021-08-13

**Authors:** Daniela Rosa Garcez, Gizele Cristina da Silva Almeida, Carlos Felipe Oliveira Silva, Tainá de Souza Nascimento, Anselmo de Athayde Costa e Silva, Ana Francisca Rozin Kleiner, Givago da Silva Souza, Elizabeth Sumi Yamada, Bianca Callegari

**Affiliations:** 1 University Hospital Bettina Ferro de Souza, Federal University of Pará, R. Augusto Corrêa, n1, Belém, Pará 66075‑110 Brazil; 2 Neuroscience and Cell Biology Graduate Program, Federal University of Pará, R. Augusto Corrêa, n 1, Belém, Pará 66075‑110 Brazil; 3 Laboratory of Human Motricity Sciences, Federal University of Pará, Av. Generalíssimo Deodoro 01, Belém, Pará 66050‑160 Brazil; 4 Tropical Medicine Center, Federal University of Pará, Av. Generalíssimo Deodoro 92, Belém, Pará 66050‑240 Brazil; 5 Master’s Program in Human Movement Sciences, Federal University of Pará, Av. Generalíssimo Deodoro 01, Belém, Pará 66050‑160 Brazil; 6 Department of Physiotherapy, Federal University of São Carlos, Rodovia Washington Luiz km235, caixa postal 676, São Carlos, São Paulo 13565‑905 Brazil; 7 Graduate Program in Medical Sciences and Oncology, Federal University of Pará, Rua dos Mundurucus 4487, Belém, Pará 66073‑005 Brazil; 8 Instituto de Ciências da Saúde, Avenida Generalíssimo Deodoro, nº1, Belém, Pará 66055‑240 Brazil

Correction to: *Scientific Reports* 10.1038/s41598-021-83837-2, published online 26 February 2021

The original version of this Article contained errors.

In the Abstract,

“This indicates that CLBP elderly patients have impairments to recover their postural control and less efficient anticipatory adjustments during the compensatory phase.”

now reads:

“This indicates that CLBP elderly patients have impairments to recover their postural control and less efficient anticipatory adjustments.”

Additionally, in Figure 4, the colours used in the key for “LBP” and “Control” were incorrectly swapped.

The original Figure 4 and accompanying legend appear below.

**Figure 4:**
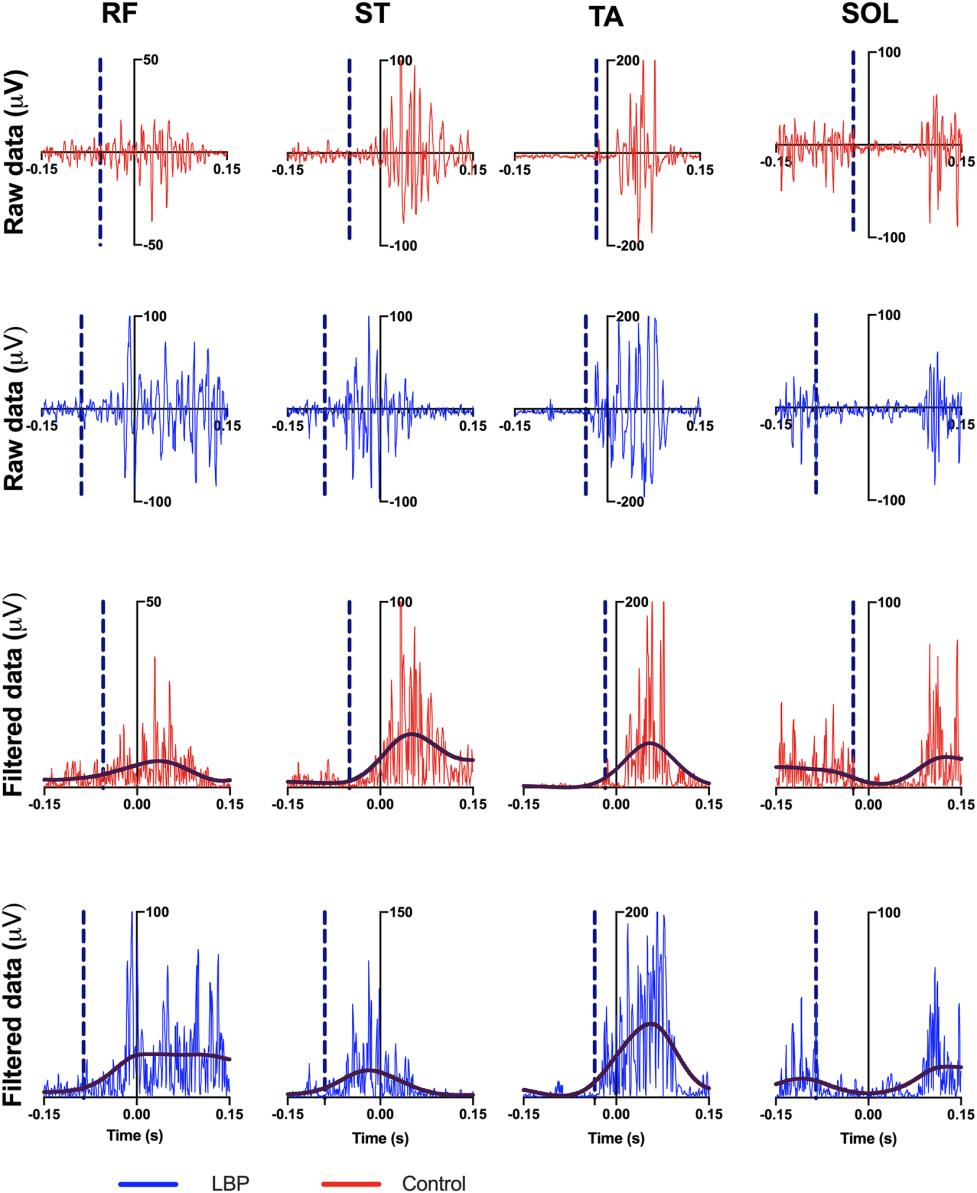
Raw and rectified 6 Hz low-pass filtered muscles activity of a typical participant of each group, recorded during one single trial. Vertical blue dashed line indicates muscles onset (t_0_). Muscle abbreviations: *ST* semitendinosus, *RF* rectus femoris, *SOL* soleous, *TA* tibialis anterior. Control participants’ anticipation compared with CLBP results.

The original Article has been corrected.

