# Supplementary material for: Postural adjustments impairments in elderly people with chronic low back pain
Source: Sci Rep. 2021 Feb 26;11:4783. doi: 10.1038/s41598-021-83837-2 (PMC7910566; doi:10.1038/s41598-021-83837-2)
Supplement: Supplementary file 1 — Supplementary Legend. [file 41598_2021_83837_MOESM1_ESM.docx]

**Supplementary Files Legend**

Datasheet used for all analysis.
